# Sequence features of *HLA-DRB1 *locus define putative basis for gene conversion and point mutations

**DOI:** 10.1186/1471-2164-9-228

**Published:** 2008-05-19

**Authors:** Jenny von Salomé, Jyrki P Kukkonen

**Affiliations:** 1 University of Helsinki, Department of Basic Veterinary Sciences, Helsinki, Finland; 2 Karolinska University Hospital, Department of Clinical Genetics, Stockholm, Sweden; 3 Åbo Akademi University, Department of Biology, Turku, Finland; 4 Uppsala University, Department of Neuroscience, Physiology, Uppsala, Sweden

## Abstract

**Background:**

HLA/MHC class II molecules show high degree of polymorphism in the human population. The individual polymorphic motifs have been suggested to be propagated and mixed by transfer of genetic material (recombination, gene conversion) between alleles, but no clear molecular basis for this has been identified as yet. A large number of MHC class II allele sequences is publicly available and could be used to analyze the sequence features behind the recombination, revealing possible basis for such recombination processes both in HLA class II genes and other genes, which recombination acts upon.

**Results:**

In this study we analyzed the vast dataset of human allelic variants (49 full coding sequences, 374 full exon 2 sequences) of the most polymorphic MHC class II locus, *HLA-DRB1*, and identified many previously unknown sequence features possibly contributing to the recombination. The CpG-dinucleotide content of exon 2 (containing the antigen-binding sites and subsequently a high degree of polymorphism) was much elevated as compared to the other exons despite similar overall G+C content. Furthermore, the CpG pattern was highly conserved. We also identified more complex, highly conserved sequence motifs in exon 2. Some of these can be identified as putative recombination motifs previously found in other genes, but most are previously unidentified.

**Conclusion:**

The identified sequence features could putatively act in recombination allowing either less (CpG dinucleotides) or more specific DNA cleavage (complex sequences) or homologous recombination (complex sequences).

## Background

Over the last few years our knowledge of the mechanism of recombination has increased substantially. Still, the knowledge is to a large extent based on simple organisms such as E. coli and yeasts, as the vertebrate genome is not equally readily or rapidly monitored or manipulated. It is well known that homologous pairing and strand exchange involved in recombination in the eukaryotic cell is promoted by specific recombination proteins [1], and that recombination is tightly linked to DNA replication and repair. For example, double strand breaks are repaired by recombination using information from homologous DNA molecules. Moreover, stalled replication can be re-started by forming a recombination intermediate with assistance from recombination proteins at the replication fork [2]. Recombination also generates diversity essential for, e.g., the vertebrate adaptive immune system (immunoglobulins and T-cell receptor genes) and long-term genome evolution. The term illegitimate recombination was coined to describe one type of "novel" recombination, which, in contrast to the classical (homologous) recombination, requires no or only short stretches of sequence homology [reviewed in [3-5]]. Despite recent advances in the investigation of eukaryotic recombination, little is known about the mechanisms of illegitimate recombination, except for some specific cases like the immunoglobulin gene rearrangements.

The major histocompatibility complex (MHC) class II loci encode heterodimeric cell surface receptors that present peptide antigens to helper T-cells so that an appropriate immune response can be induced. In man, the by-far most polymorphic MHC class II locus is *HLA-DRB1*; as of march 2008 the *HLA-DRB1 *locus had over 540 alleles [6,7] and is thus one of the most polymorphic loci in the human genome. A large number of low-frequency alleles is apparently maintained in the human population by balancing selection. The peptide fragments are bound by interactions with the peptide backbone and amino acid side chains in the second exon-coded part of *HLA-DRB1 *(*DRB1*-e2), termed antigen recognition sites (ARS). Each individual carries a maximum number of two different inherited alleles per locus (assuming heterozygocity), while the greater allelic diversity is present in the population, putatively allowing population adaptation to pathogens.

ARS polymorphisms are thought to be created by point mutations, which are propagated by some recombination events, e.g. gene conversion. This view is based on the observed patchwork pattern of apparently exchanged motifs and the fact that synonymous substitutions are also much elevated in the *DRB1*-e2 (hitch-hiking with the non-synonymous substitutions) [8-11]. However, there is little direct evidence for any recombination in MHC class II ARS, and no clear recombinogenic motifs or mechanisms have as yet been identified. Since the multiple ARS of *DRB1*-e2 are spread over a small region of 200 bp only, exchange of very small blocks of DNA is needed to create the pattern of polymorphism seen. This, again, is in sharp contrast to the classical (homologous) recombination, which requires significant stretches of sequence homology and exchanges relatively large blocks of generic material. Therefore, due to the apparent high activity of illegitimate recombination in *DRB1*-e2 and the large number of allelic sequences known, *DRB1*-e2 seems to be a uniquely suitable target for investigations of mechanisms behind illegitimate recombination. As it is known that specific DNA sequences can enhance or mediate recombination, we have in this study targeted the vast database of known human *HLA-DRB1 *alleles in the quest for possible sequence motifs that would enable recombination. The analyses identify strongly conserved sequence features as well as recombinogenic motifs previously recognized in other genes, which may thus lie at the basis of recombination events creating new alleles.

## Results

### Diversity in the antigen-binding exon

**Figure 1:**
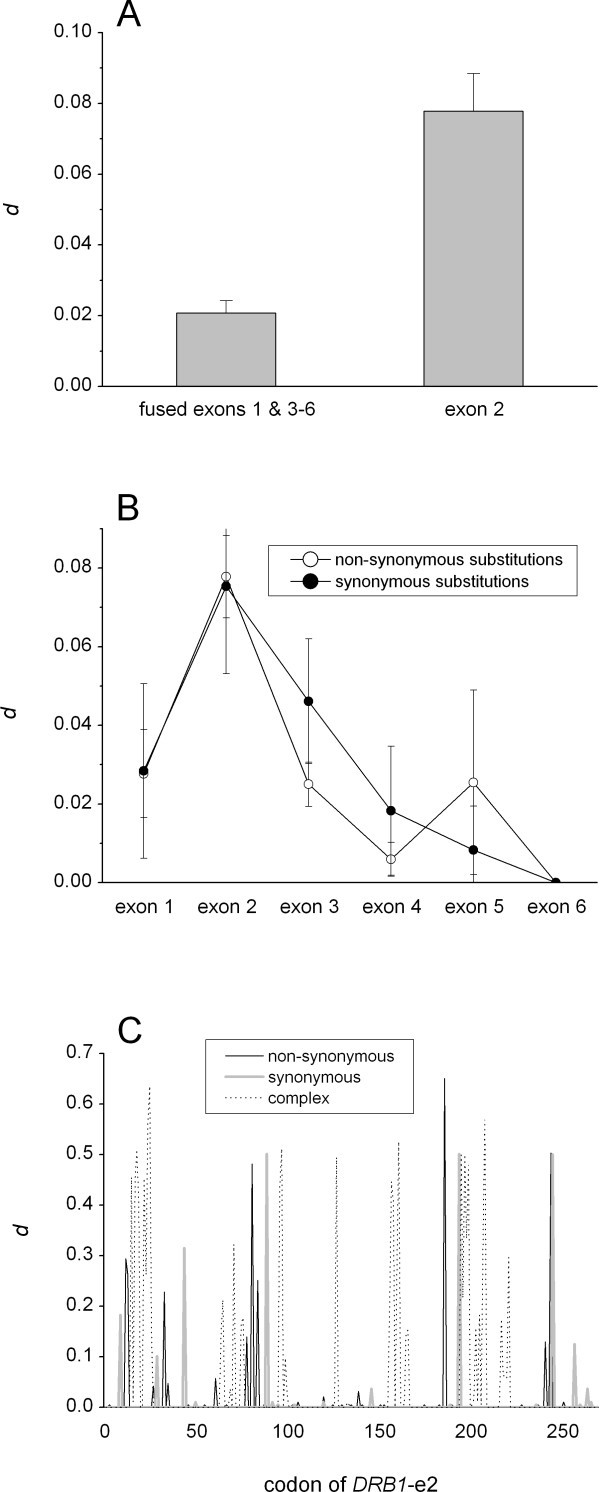
The *HLA-DRB1 *exon diversity. A, *DRB1 *exon 2 diversity compared to the rest of the coding region (fused exons 1, 3, 4, 5 and 6) in the dataset including the entire *DRB1 *coding region (49 sequences). Mean ± sem is shown. B, synonymous and non-synonymous diversity in the *DRB1 *coding region in the dataset including the entire *DRB1 *coding region. In the short exon 5 (24 bp) half of the alleles have G instead of C at the nucleotide position 22, resulting in high apparent diversity for the whole exon. Mean ± sem is shown. C, sliding window analysis of non-synonymous, synonymous and complex substitutions in the *DRB1*-e2 in the dataset including the complete *DRB1*-e2. Complex stands for complex combinations of non-synonymous and synonymous substitutions in the same codon. The graph illustrates the contribution of these different components in *d*, which is not equal to *d*_*synonymous *_and *d*_*non-synonymous *_(*d*, as calculated here does not take into consideration the capability of the codon to mutate in synonymous and non-synonymous manner).

*DRB1*-e2 displays much higher degree of sequence diversity than the other exons in *DRB1 *(Fig. 1A), independently of whether gross-diversity or non-synonymous (aminoacid-changing) diversity is analyzed [12]. As seen in earlier studies [see e.g. [12]], the synonymous diversity is also elevated in exon 2 (Fig. 1B), supporting the view that recombination, e.g. gene conversion is involved in creating polymorphism in this exon [13,14,10]. Consequently, synonymous substitutions would "hitch-hike" within the same exchanged DNA blocks as the non-synonymous substitutions and remain conserved due to selection forces acting on the non-synonymous substitutions. Indeed, synonymous substitutions were mainly found either in the same codons as the non-synonymous ones (Fig. 1C, the "complex" trace in its entity and the overlap of the non-synonymous and synonymous traces) or in their close vicinity (Fig. 1C).

### Frequency of transitions and transversions in the coding region

**Figure 2:**
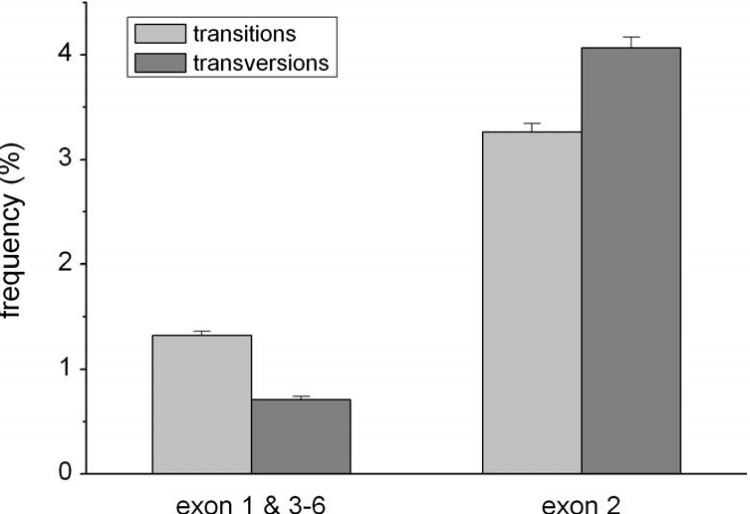
Transitions and transversions in *HLA-DRB1*, based on the dataset including the entire *DRB1 *coding region (49 sequences). Mean ± sem is shown.

The higher diversity of *DRB1*-e2 is also reflected by a higher frequency of both transitions (T↔C, A↔G) and transversions (T↔A, T↔G, C↔A, C↔G), as compared to the rest of the *DRB1 *exons (Fig. 2). In general, transitions occur at higher frequencies than transversions in our genome [15]. However, while the transition/transversion-ratio was about 2 in the fused other exons (between 2 and 3 in the separate exons 1, 3 and 4, which have a length comparable to exon 2), it was 0.8 in exon 2. The results using the full dataset of 374 complete *DRB1*-e2 (transitions = 3.4 ± 0.0%, transversions = 4.2 ± 0.1%, ratio = 0.8) were similar to the 49 complete coding sequences (Fig. 2). The transition/transversion ratio near unity in *DRB1*-e2 is logical in the light of previous studies, which show that when sequences diverge and mutations accumulate, the transition/transversion-ratio decreases finally approaching 1 due to transition saturation [16,17].

### CpG dinucleotide enrichment and conservation in DRB1-e2

**Figure 3:**
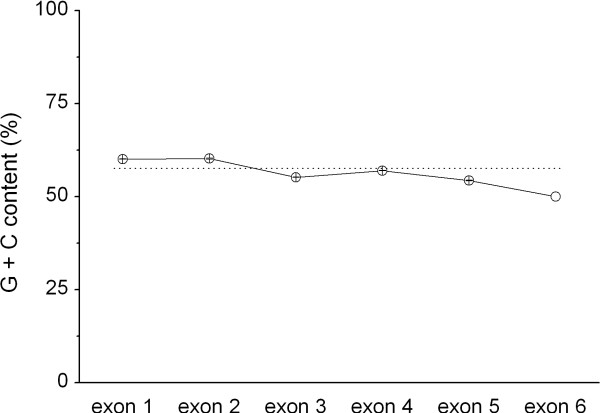
G+C content in *HLA-DRB1 *exons 1–6, based on the dataset including the entire *DRB1 *coding region (49 sequences). The dotted line indicates the overall average G+C. Mean ± sem is shown.

The G+C level was similar across all *DRB1 *exons (Fig. 3). At the determined G+C content of the whole of *DRB1 *of 60%, the theoretical level of CpG should under neutral conditions be 9% (see below). When present in the CpG-dinucleotide, cytosine is often methylated. Methyl-cytosine can then deaminate to uracil, and thus lead to the transition C→T or, if occurring in the complementary strand, a G→A transition. Thus, CG may mutate to TG or CA, and genes regularly have a lower than mathematically expected level of CpG dinucleotides. The high propensity of CpG to mutate may also effectively engage DNA repair. DNA repair induces double strand breaks and may support recombination events, which may explain why CpG-rich sequences have been identified to display high recombination activity.

**Figure 4:**
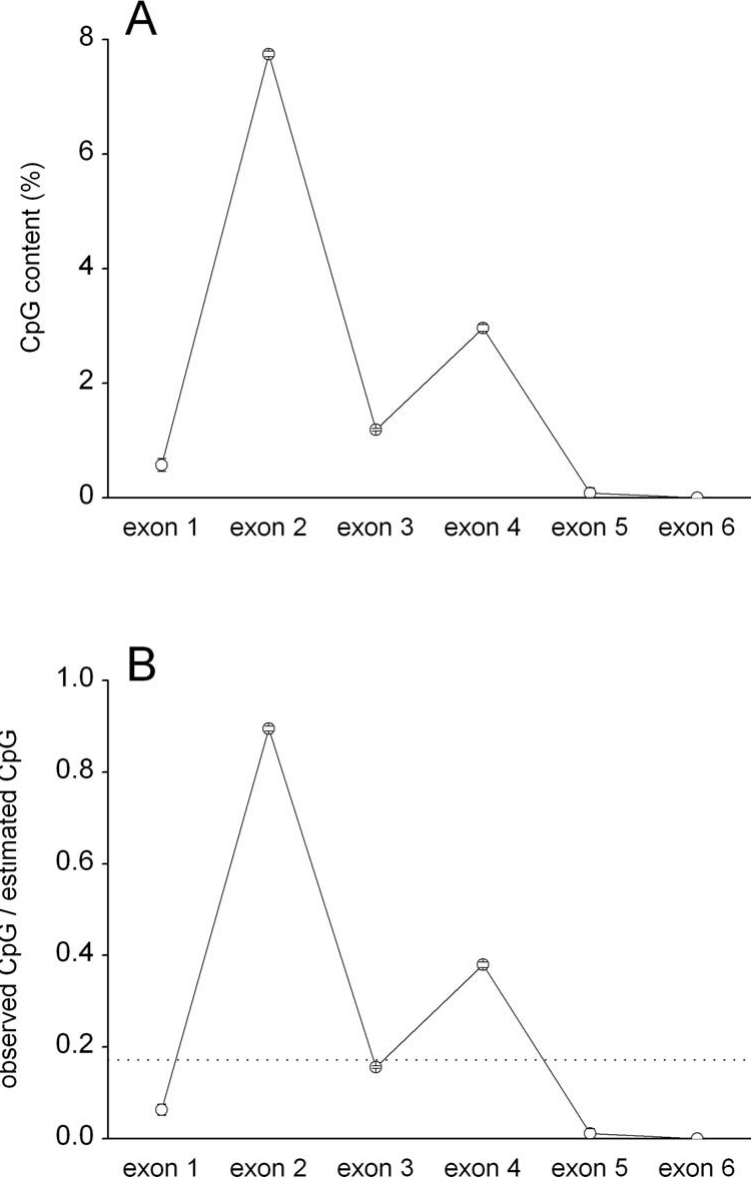
CpG-dinucleotide content in *HLA-DRB1 *exons 1–6, based on the dataset including the entire *DRB1 *coding region (49 sequences). A, the observed CpG-dinucleotide content. B, the observed CpG level (as in A) divided by the mathematically estimated CpG content (based on the total G+C level). Mean ± sem is shown. The ratios were separately calculated for each allele and then averaged.

**Figure 5:**
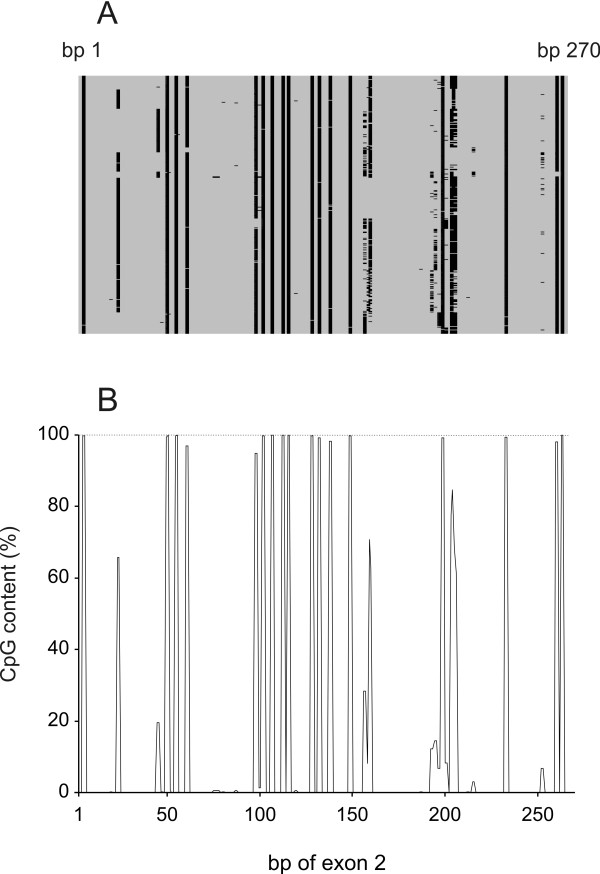
CpG distribution in *DRB1*-e2, based on the dataset including the 374 complete *DRB1*-e2 sequences. A, each individual sequence lined under each other in the consensus numbering order starting from DRB1*010101. Black boxes indicate CpG dinucleotides and gray boxes other dinucleotides. B, CpG frequency for each nucleotide position. The dotted line indicates 100%.

Theoretically, CpG content = ((G+C content/100%)/2)^2 ^* 100% (e.g. for G+C content of 50%, CpG content = 6.25%). The actual CpG content depends on the age of the gene, but in average the content would be below one third of the theoretical (e.g. for G+C content of 50%, CpG content < 2%) [18]. As expected, the determined CpG content of *DRB1*-e1, and -3-6 was well below the theoretical level of 9% (Fig. 4A; in average ~1/6 of the theoretical level [dotted line in Fig. 4B]). In contrast, CpG content of *DRB1*-e2 was surprisingly high, about 8% (Fig. 4A), which suggests that the CpG level is almost fully preserved in *DRB1*-e2 (see also Fig. 4B). In addition, the distribution of CpG dinucleotides in *DRB1*-e2 was to a very high extent conserved in all alleles (Fig. 5AB).

### Motifs potentially involved in site-specific recombination

There is a number of sequence features or motifs proposed to be recognized by specific nuclease complexes, resulting in double strand breaks and increased recombination rate [19,20]. We further analyzed the sequence data sets to explore the possibility that specific recombination motifs are involved in creating polymorphism in *DRB1*-e2 (Table 1).

**Table 1:** Motifs used in the screening of *DRB1*-e2.

**Motif description**	**Motif sequence**	**Reference**
Polypurine/-pyrimidine tract	5'-RRRRR-3'/5'-YYYYY-3'	[47, 19, 48, 49]
Alternating purine-pyrimidine tract	5'-RYRYR-3'/5'-YRYRY-3'	[19, 50]
Immunoglobulin heavy chain class switch repeats	5'-GAGCT-3'/5'-AGCTC-3'	[51, 49]
	5'-GGGCT-3'/5'-AGCCC-3'	
	5'-GGGGT-3'/5'-ACCCC-3'	
	5'-TGGGG-3'/5'-CCCCA-3'	
	5'-TGAGC-3'/5'-GCTCA-3'	
DNA polymerase arrest site	5'-WGGAG-3'/5'-CTCCW-3'	[49]
Deletion hotspot consensus	5'-TGRRKM-3'/5'-KMYYCA-3'	[28, 49]
Heptamer recombination signal	5'-CACAGTG-3'/5'-CACTGTG-3'	[22]
Nonamer recombination signal	5'-ACAAAAACC-3'/5'-GGTTTTTGT-3'	[22]
Chi-like sequence	5'-GCTGGGG-3'/5'-CCCCAGC-3'	[40, 52]
Chi-like sequence	5'-CCAG-3'/5'-CTGG-3'	[53, 54]
Chi-like sequence	5'-GCWGGWGG-3'/5'-CCWCCWGC-3'	[55]
Topoisomerase I consensus cleavage sites	5'-CAT-3'/5'-ATG-3'	[56]
	5'-CTY-3'/5'-RAG-3'	
	5'-GTY-3'/5'-RAC-3'	
DNA polymerase A pause site core sequence	5'-GAG-3'/5'-CTC-3'	[57]
	5'-ACG-3'/5'-CGT-3'	
DNA polymerase A/B frameshift hotspots	5'-TGGNGT-3'/5'-ACNCCA-3'	[58, 59]
Vertebrate topoisomerase II consensus cleavage site	5'-RNYNNCNNGYNGKTNYNY-3'/	[60, 61]
	5'-RNRNAMCNRCNNGNNTNY-3'	
Human hypervariable minisatellite core sequence	5'-GGGCAGGANG-3'/5'-CNTCCTGCCC-3'	[62]
DNA polymerase A frameshift hotspots	5'-TCCCCC-3'/5'-GGGGGA-3'	[59, 63]
DNA polymerase B frameshift hotspots	5'-TTTT-3'/5'-AAAA-3'	[58]
Indel hotspot	5'-GTAAGT-3'/5'-ACTTAC-3'	[64]
Hotspot motif	5'-CCTCCCT-3'/5'-AGGGAGG-3'	[63]
Repeat element motif	5'-CCCCACCCC-3'/5'-GGGGTGGGG-3'	[63]
Double strand break-generating motif	5'-TGGGGG-3'/5'-CCCCCA-3'	[63]

The sequences of the complementary strands are separated by "/". The ambiguity code symbols are: R = A/G, Y = C/T, K = G/T, M = A/C, S = G/C, W = A/T, N = A/C/G/T.

Recombination signal sequences (RSSs) are involved in the diversification of antibody genes, initiated by DNA double-strand breaks introduced in the vicinity of RSSs. The RSSs are composed of conserved heptamer and nonamer motifs separated by a spacer of 12 or 23 bp [21]. The heptamer motifs, especially the first three bases (CAC), are the most influential on recombination efficiency and are usually the most conserved [22]. We found a heptamer-like motif (5'-CAC**G**GTG-3', the bold letter is replaced in 7% of the alleles) at position 254–260 in exon 2.

Class switch recombination (CSR) refers to the event when a lymphocyte changes the type of immunoglobulin it produces [23]. Also CSR involves recombination via DNA double strand breaks at switch regions containing repetitive elements (predominantly, 5'-GAGCT-3' and 5'-GGGGT-3'). The immunoglobulin heavy chain class switch repeat GAGCT was present at position 145–149 and 248–252 in all but one (DRB1*0423) of 374 *DRB1 *alleles (Table 2). Another immunoglobulin heavy chain class switch repeat (5'-TGGGG-3') was present in all alleles except for alleles in the lineage DRB1*07 (Table 2). This immunoglobulin heavy chain class switch repeats was also present in the exon 2 sequences excluded from the analysis due to missing bases in either the 3' or 5' end (see Additional file 1).

**Table 2:** Motifs previously identified in other genes found in the *DRB1*-e2

**Motif description**	**Motif sequence**	**Position**	**Not present in**
Polypurine tract	5'-RRRRR(RRR)-3'	88–95	
		121–125^a^	
		178–184	
		246–250	
Polypyrimidine tract	5'-YYYYY-3'	5–10	
		36–41	
Immunoglobulin heavy chain class switch repeat	5'-GAGCT-3'	141–145	DRB1*0328
		248–252	DRB1*0452
	5'-TGGGG-3'	145–149	DRB1*07
Deletion hotspot consensus	5'-TGAAGA-3'	37–42^b^	
	5'-TGRRKM-3'	145–150	DRB1*07
		250–255^b^	
Chi-like sequence	5'-GCTGGGG-3'	143–149	DRB1*07
	5'-CTGG-3'	144–147^b^	DRB1*07
		167–170^b^	DRB1*0705
		176–179	
Topoisomerase I consensus cleavage site	5'-CTY-3'	38–40	
		251–253	DRB1*0423, *0452
	5'-GTY-3'	4–6	
		31–33	
		47–49^b^	
		108–110^b^	
		114–116^b^	
		171–173^b^	
		183–185^b^	
		213–215^b^	
		231–233^b^	
DNA polymerase a pause site core sequence	5'-GAG-3'	51–53	DRB1*0439
		121–123	
		246–251 (2×)	
		268–270	
	5'-ACG-3'	2–4	
		115–117	
		116–118^b^	
Deletion hotspot	5'-YYYTG-3'	7–11	
		177–181^b^	DRB1*0705
		187–191	DRB1*1374

Only the motifs present in at least an (almost) entire allelic family are presented; some less common motifs are presented in the text. The ambiguity code symbols are: R = A/G, Y = C/T, K = G/T, M = A/C, S = G/C, W = A/T, N = A/C/G/T.

^a^motif ± 1 bp

^b ^motif in non-coding strand corresponding to these bases in the coding strand.

Chi (crossover hotspot instigator, χ) is an octamer recombination hotspot (5'-GCTGGTGG-3') of the major recombination pathway in *E. coli *[reviewed in [24]]. Recombination by this pathway is initiated by double-strand breaks occurring at chi sequences. Variants of this motif are suggested to have partial recombinogenic activity, and chi-like sequences have been speculated to be involved in both deletion and translocation events in man [25-27]. A chi-like sequences at nucleotide position 143–149 in exon 2 was found in all alleles except for the DRB1*07 allelic lineage (Table 2). The chi-like sequence in *DRB1*-e2 was overlapping with a motif reported to be a deletion hotspot consensus sequence (5'-TGRRKM-3'), suggested to be involved in illegitimate recombination [28,29]. We located this hotspot sequence at positions 145–150 in all alleles except for the allelic lineage DRB1*07 (Table 2). Moreover, this sequence was present in the non-coding strand of all alleles at coding strand position 37–42 (Table 2).

Several types of the recombination motifs screened for were also found in the other exons of *DRB1 *(the dataset of 49 complete coding sequences) (not shown).

### Conserved sequence stretches and motifs in DRB1-e2

**Figure 6:**
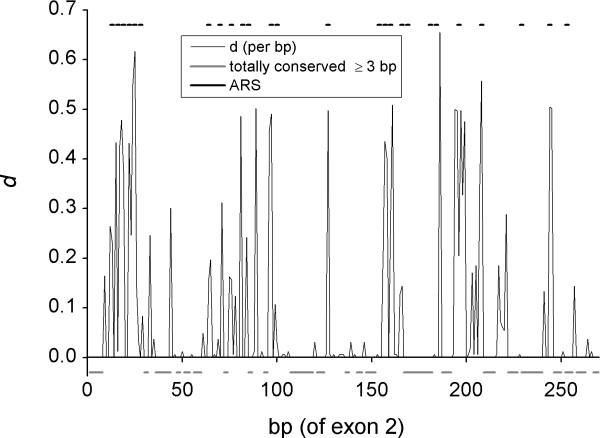
Sliding window analysis of nucleotide diversity in *HLA-DRB1 *exon 2, displaying stretches of totally conserved bases in the 374 *DRB1*-e2 sequences (of the length ≥ 3 bp; thick grey lines below the abscissa). Also indicated are the previously identified ARS-coding codons (thick black lines above the diversity graph).

Despite the high degree of variability in *DRB1*-e2 we could, to our surprise, find 19 stretches of a length 3–13 bp that have no variation at all among different *DRB1*-e2 (Table 3 and Fig. 6). Some of these fully conserved bases corresponded to the known motifs as identified above (Table 3).

**Table 3:** Fully conserved stretches of a minimum of 3 bp in all *DRB1*-e2 sequences

**Position**	**Sequence (underlined letters corresponding to motif in table 2)**	**Corresponding motif in Table 2**
4–8	5'-GTTTC-3'	Polypyrimidine tract
30–32	5'-TGT-3'	
36–43	5'-TTCTTCAA-3' 5'-TTCTTCAA-3'	Deletion hotspot consensus sequence (5'-TGAAGA-3') in non-coding strand Polypyrimidine tract (5'-TTCTTC-3')
47–49	5'-GAC-3'	
51–54	5'-GAGC-3'	
56–60	5'-GGTGC-3'	
107–119	5'-CGACAGCGACGTG-3'	
121–124	5'-GGGA-3'	Polypurine tract
142–145	5'-AGCT-3'	Part of the immunoglobulin heavy chain class switch repeat (5'-GAGCT-3')^a^
	5'-AGCT-3'	Part of the chi-like sequence (5'-GCTGGGG-3')^a^
147–149	5'-GGG-3'	Part of deletion hotspot consensus sequence (5'-TGRRKM-3')^a^
	5'-GGG-3'	Part of the chi-like sequence (5'-GCTGGGG-3')^a^
167–174	5'-CTGGAACA-3'	
179–182	5'-GAAG-3'	
210–215	5'-GTGGAC-3'	
222–227	5'-TGCAGA-3'	
229–235	5'-ACAACTA-3'	
246–250	5'-GAGAG-3'	Polypurine trac
248–250	5'-GAG-3'	Part of the immunoglobulin heavy chain class switch repeat (5'-GAGCT-3')^a^
252–256	5'-TTCAC-3'	Deletion hotspot consensus sequence
261–263	5'-CAG-3'	
267–269	5'-CGA-3'	

^a^the full motif as in Table 2

## Discussion

In this study we identify several distinct features of exon 2 of *DRB1*. One of these is its high CpG content, possibly leading to a high degree of a) point mutations and b) DNA repair. However, not only is the CpG level high in *DRB1*-e2, but also the CpG pattern is highly conserved in *DRB*-e2. It therefore appears unlikely that CpG-dinucleotides would support ARS polymorphism by point mutations. More likely is that the conserved CpG pattern is explained by frequent DNA repair, which, by introducing double-strand DNA cleavage followed by non-homologous end-joining, is one of the suggested mechanisms of gene conversion [reviewed in [3,5,30]]. Earlier studies of MHC class I nucleotide sequences in mice have proposed that regions with high levels of CpG dinucleotides are involved in non-reciprocal recombination (gene conversion) [31]. Analyzes of human MHC class I and II sequences also have reported increased CpG dinucleotide levels in regions suggested to be involved in gene conversion [32]. CpG nucleotide could be preserved if the repair system had a bias towards G:C pairs instead of A:T pairs [33] as suggested for regions with high recombination activity [34]. However, it should be born in mind that unmethylated CpG dinucleotides, in contrast to the cytosine-methylated CpG:s, mutate at normal rates and regions with high CpG contents may have low levels of methylation [35]. Conservation of CpG dinucleotides may therefore be a result of either low germ-line methylation or a specific selection against the loss of CpGs [36]. However, the highly significant pattern of conserved CpG in *HLA-DRB1-e2 *can be considered unlikely even if the CpG dinucleotides were unmethylated and mutated at the rate of other bases.

A few eukaryotic endonucleases with specific DNA recognition sequences involved in DNA recombination, such as topoisomerase I [37], Endo.SceI [38] and homing endonucleases [reviewed in [39]], have been identified. The enzymes have in common that they recognize a more or less strictly defined DNA sequence and cleave at it or some distance from it. In addition, a number of other conserved sequence motifs associated with high recombination activity (such as the chi-like sequences) but without a pinpointed endonuclease/recombinase have been recognized [40,19,41]. In this study, we screened *DRB1*-e2 for "known" recombination, translocation and deletion motifs. A heptamer-like motif was found in all investigated *DRB1 *alleles and an immunoglobulin heavy chain class switch repeat was found in all but one of the *DRB1 *alleles. Moreover, a chi-like sequence and a deletion hotspot consensus sequence were present in all alleles except for the *07 lineage. It is thinkable that the DRB1*07 allelic lineage, which contains least alleles of all the lineages, may have lost one of these motifs and therefore gained a limited ability to recombine. This may also be true for other, even less frequent motifs, also including conserved CpGs. Currently, not enough is known about the function of the specific motifs found in order to speculate further on their function. Interspersed among the highly polymorphic areas of *DRB1*-e2, we found multiple short stretches of bases that have no variation at all between the *DRB1 *alleles in the dataset. Conserved amino acid motifs can be important for the maintenance of the overall structure of the antigen-binding groove, but as these stretches also lack synonymous substitutions they may have a function in allowing recombination between alleles via illegitimate recombination. This could occur either by offering homology for recombination, by allowing cleavage by some specific enzymes or by stabilization of DNA's secondary structure. Comparison of these sequences to known sequence motifs associated with recombination (see above) produced no hits, which is by no means surprising as indeed only few motifs are known and even fewer verified.

It should be born in mind that the specific sequence motifs screened for are for the most part very short and may thus appear in a random fashion in any sequence analyzed. Indeed, some motifs were found in the other exons of *HLA-DRB1*, not subject to recombination. However, the fact that exon 2 is subject to high rate of recombination – in contrast to the other exons which are highly conserved – makes random conservation of such stretches unlikely, especially in such a large pool of allelic sequences. Yet the most remarkable features of *DRB1*-e2 are, rather than the known recombinogenic motifs found, a) the fully conserved sequence stretches and b) high CpG content and the conserved CpG pattern.

## Conclusion

We have identified in *DRB1*-e2 both some known recombination motifs and multiple putative motifs. The latter include both the conserved CpG pattern and other fully conserved sequence motifs. Although the role of these sequence features in the recombination processes in *DRB1 *is speculative, it is obvious that the known recombination motifs identified here cannot be enough to support the full spectrum of recombination. 22 variable and 15 conserved *DRB1*-e2 ARS-coding codons, spread over 245 bp (Fig. 6), are known, and each of the variable ARS codons should probably be able to recombine separately from the others, theoretically requiring 23 recombination breakpoints. Whether this indeed is the case, will be deduced from full mapping of the *DRB1*-e2 recombination profile, which is currently in progress. If the conserved sequence motifs identified here indeed are important in recombination, they would likely be present in other regions of the genome with high recombination activity. This will also be addressed in future studies.

## Methods

### Nucleotide sequences used

For the analysis, sequences from the IMGT/HLA database [6,7] were used. The datasets analyzed were the 374 complete exon 2 sequences and 49 complete coding sequences (exons 1–6). Full descriptions of the datasets can be found in the Additional files 2 and 3.

### Analysis of diversity, transition/transversion-ratios, G+C and CpG contents

The sequences were aligned using ClustalW [42]. The mean synonymous and non-synonymous diversities (*d*) were estimated by pairwise comparison of the number of nucleotide substitutions using the Jukes-Cantor method [43] with the MEGA3.1 software [44]. Sliding-window analyses of the nucleotide diversities were performed using DnaSP 4.10.9 [45] Analyses of the transition/transversion-ratios and the G+C and CpG contents were done with SWAAP 1.0.2 [46] and MEGA3.1. The sliding window analyses of the CpG content were performed using Microsoft Excel.

### Analysis of motifs potentially involved in site-specific recombination

Recombination has been suggested to be promoted by common sequence features or motifs [20], known or postulated to be recognized by specific nuclease complexes, leading to double strand break and increased recombination rate. We screened *DRB1*-e2 (coding and non-coding strands) in MEGA3.1 for sequence motifs previously shown to be involved in recombination, to explore the possibility that specific motifs are involved in creating new polymorphisms. The motifs screened for are listed in Table 1.

## List of abbreviations

ARS: antigen-recognition site(s); bp: basepairs; Chi: crossover hotspot instigator; CpG: CG-dinucleotide (in DNA); CSR: class switch recombination; *d*: nucleotide diversity; G+C content: content of guanine and cytosine nucleotides (in DNA), *DRB1*-e2: exon 2 of the *HLA-DRB1 *gene; HLA: human leukocyte antigen; MHC: major histocompatibility complex; RSSs: recombination signal sequences.

## Authors' contributions

JPK designed the study, JvS and JPK performed the analyses and wrote the manuscript. Both authors read and approved the final manuscript.

## Supplementary Material

Additional file 1 The 98 incomplete *HLA-DRB1 *exon 2 sequences excluded from the analyses. Click here for file

Additional file 2 The 374 complete *HLA-DRB1 *exon 2 sequences used in the analyses. Click here for file

Additional file 3 The 49 complete *HLA-DRB1 *coding sequences used in the analyses. Click here for file
